# Elasticity mapping of gastrocnemius fascia and myotendinous junction of the medial gastrocnemius during passive ankle and knee dorsiflexion

**DOI:** 10.3389/fspor.2026.1797883

**Published:** 2026-04-01

**Authors:** Xingxian Situ, Jiping Zhou, Changzheng Li, Zhijie Zhang, Yuyi Lin

**Affiliations:** 1 Shenzhen Dapeng New District Nan'ao People's Hospital, branch of the First Affiliate of Shenzhen University, Shenzhen, China; 2 Department of Rehabilitation Therapy, Luoyang Orthopedics Hospital of Henan Province, Luoyang, China

**Keywords:** elasticity, fascia, imaging techniques, muscle strain, skeletal, ultrasound shear wave elastography

## Abstract

**Background and purpose:**

Muscle strain is a common side-effect of exercise. In this context, many scholars have focused primarily on the biological characteristics of the muscle, generally overlooking the importance of myofascial elasticity. Therefore, the aims of the present study were: (1) to evaluate the intra- and inter-operator reliability of elasticity measurements in the myofascial and myotendinous junction (MTJ) using ultrasound shear wave elastography (USWE); (2) to establish the association between myofascial elasticity and passive joint angles.

**Methods:**

Twenty-one healthy and physically active adults participated in this study. The elasticity of the medial gastrocnemius muscle fascia (MGF), lateral gastrocnemius muscle fascia (LGF), and MTJ were quantified at different angles using USWE.

**Results:**

USWE demonstrated excellent intra-operator (ICC = 0.893–0.980) and inter-operator (ICC = 0.907–0.981) reliability for quantifying the elastic modulus of the gastrocnemius fasciae and MTJ. The elasticity of these structures was significantly influenced by joint angles: it decreased with knee flexion and increased with ankle dorsiflexion (*p* < 0.05). Across all postures, the MTJ exhibited consistently higher elasticity than the fascial layers (*p* < 0.001). When the knee joint was flexed at 90°, there was no significant difference in the elasticity of the MGF compared to the LGF, regardless of the ankle joint angle (*p* > 0.05). However, when the knee joint was fully extended, the elasticity of the LGF at plantar flexion (PF) angles of 0° and 50° was significantly greater than that of the MGF (*p* < 0.01), while the elasticity was higher in the MGF than in the LGF at dorsiflexion (DF) 20° (*p* < 0.01).

**Conclusions:**

This study confirms the high reliability of USWE for assessing myofascial elasticity and reveals its dynamic regulation by joint posture. The findings provide crucial imaging-based biomechanical evidence that enhances the understanding of myofascial mechanics *in vivo*, which may guide future evaluation and rehabilitation strategies for muscle strain-related injuries.

## Introduction

Myofascial trigger points are important contributors to musculoskeletal discomfort and pain in many populations, including athletes (1–3). They not only alter muscular activation and movement patterns in the musculoskeletal system but also potentially accelerate muscle fatigability and increase the risk of muscle function impairments, such as muscle cramps and muscle strain (4, 5). The traditional anatomical view is that muscle architecture (e.g., the cross-sectional area of muscle, and the angles and lengths of muscle fibers) is a key determinant of muscle strength generation and effective movement production (6, 7). It usually involves isolated analyses of muscle actions, ignoring the structural continuity and functional significance of connective tissue (4, 5). Most crucially, muscle force is not completely transmitted to the tendon but also through the force transmission mechanism of myofascial elasticity. This means that force transmission is also influenced by the fascia, the aponeurosis of the myotendinous junction (MTJ), and myotendinous structures surrounding the muscle (8, 9). Against this background, it seems advisable to eliminate the latent myofascial trigger points to prevent the development of chronic pain syndromes and optimize muscle function.

Fascia, as an uninterrupted viscoelastic membranous tissue, is mainly composed of collagen fibers. However, fascia is difficult to isolate as it penetrates and surrounds all structures of the whole body (10). Fascia acts to keep continuity amongst structures to build up support and function (e.g., myofascial force transmission). Anatomical studies have demonstrated that the muscle fascia is composed of three layers of connective tissue with different orientations and densities, and it connects to the underlying muscles (11–13). These characteristics not only strengthen the ability of fascial tissues to bear strain (including the force produced by intra- and inter-muscle contraction) in all directions but also allow the transmission of forces to adjacent tendons or muscles in an effective way (14, 15). Previous studies have shown that myofascial force transmissions occur not only intra-limb but also intra-muscle. Both active muscle contraction and passive joint angle change could induce myofascial force transmission (16, 17). In the lower leg of humans, the Achilles tendon (AT) is the conjoined tendon of the triceps surae, including the soleus muscle (SOL), the lateral head of gastrocnemius (LG), and the medial head of gastrocnemius (MG). The gastrocnemius muscle spans the ankle and knee, and is thus susceptible to the movement of these two joints. In addition, the neighboring aponeuroses of SOL, MG and LG are displaced differently upon activation depending on the angle of the knee joint, which indicates that the force on the aponeurosis is different (18, 19). The altered mechanical properties of musculoskeletal tissues, aponeurosis and fascia can be the cause or consequence of several pathologies (20–22). For instance, a decrease in the elastic properties of the plantar aponeurosis may be the result of plantar fasciitis, while an increase in the elasticity of crus myofasciae is related to compartment syndrome (23, 24). Thus, quantifying the elastic properties of musculoskeletal tissues, aponeurosis and fascia could improve our ability to prevent or diagnose myofascial trigger points and myofascial pain.

Ultrasound shear wave elastography (USWE), an objective and effective technique to quantify the elastic properties of soft tissues, such as muscle and tendon, can be used to assess the elastic modulus of a local target area through shear wave speed. In allows a deeper insight into how the body responds to various forces and the effects of treatments beneath the skin. The elastic properties of soft tissues have been measured using USWE under various conditions (e.g., at rest, before and after stretching, during and after exercise) (25–27). Specifically, recent studies have robustly validated the ability of USWE to assess the gastrocnemius muscle belly. For instance, previous studies have established the reliability of SWE in assessing gastrocnemius stiffness (28) and have preliminarily explored its application under changes in a single joint angle, such as ankle dorsiflexion or plantarflexion (29, 30). In addition, research has confirmed the excellent reliability of this technique in measuring medial and lateral gastrocnemius stiffness during graded voluntary contractions (31). Furthermore, the elasticity of the Achilles tendon and its functional behavior under load has been a focus of recent studies, which demonstrated significant changes in tendon stiffness across different ankle joint angles (32). Concurrently, the functional role of fascial tissues in force transmission and injury mechanisms has received renewed emphasis in biomechanical literature (33). However, despite this progress, direct *in vivo* quantifications of the epimysial fascia enveloping the gastrocnemius muscles remain notably scarce. Existing studies on fascial elasticity often focus on other anatomical regions or are conducted in static, neutral postures (25–27). This reveals a clear gap in recent data on the dynamic elastic behavior of the medial and lateral gastrocnemius fasciae (MGF, LGF) and their MTJ in response to systematic, passive changes in both the knee and ankle angles. In specific, the precise biomechanical relationship governing how elasticity in these specific, interconnected structures changes with combined joint postures is yet to be elucidated.

In summary, this study evaluates the intra- and inter-operator reliability of elasticity measurements in the myofascial and MTJ using USWE, and explores the effect of passive joint angle on myofascial elastic properties in lower limbs. To achieve a clear knowledge of the mechanical properties of normal myofasciae, changes in the different angles of the ankle joint, with the knee flexed 90° or fully extended on the elastic properties of the MGF, LGF and MTJ, are investigated. It is hypothesized that the muscle fascia and MTJ become stiffer at greater stretch angles, and both ankle dorsiflexion and knee flexion induce non-homogeneous changes in myofascial and MTJ elasticity.

## Materials and methods

### Ethics considerations

This study was approved by the Ethics committee of Shenzhen Dapeng New District Nan'ao People's Hospital, Shenzen, China (No. 20230301009). All participants were fully informed of matters related to this study and signed a written informed consent. Sample size was determined via a power analysis (G* Power software, version 3.1.9.2) based on prior study (26). The significance level (alpha) was established at 0.05, and the statistical power was set at 0.80.

### Participants

Twenty-one male physically active adults (age: 21.67 ± 1.06 years, height: 1.72 ± 0.05 m, body mass: 61.45 ± 5.66 kg, BMI: 20.88 ± 1.84 kg/m^2^) without any history of lower limbs injury were invited to participate. All participants were required to have at least 20° of passive ankle dorsiflexion and 50° of passive ankle plantarflexion to ensure they could achieve all prescribed testing postures. This study was performed at Nanao People's Hospital, Dapeng New District, Shenzhen, China. Each enrolled physically active adult was engaged in over 300 min of moderate-intensity aerobic exercise or in over 150 min of vigorous-intensity aerobic exercise per week (34).

### Ultrasound shear wave elastography

The procedures for muscle fascia and MTJ elasticity measurement were similar to those in our previous studies (25–27). The equipment consisted of an ultrasonic instrument (Aixplorer Supersonic Imagine, Aix-en-Provence, France) with built-in shear wave elastic imaging technology. A 40 mm linear-array transducer (SL15-4, Supersonic Imagine, France) was employed to capture USWE ultrasound images and quantify the elasticity of MGF, LGF and MTJ. The settings of the AixPlorer ultrasonic scanner were as follows: the shear wave elastography mode was musculoskeletal mode; the frequency was 4–15 MHz; USWE Options was in penetration mode; the opacity was 85%; the shear modulus range was 0–200 kPa; the B-scan depth was 3.0 cm (26). The Q-box diameter of MGF, LGF and MTJ were set as 1 mm (25–27). To gain accurate values of passive tension, the ROI was set as large as possible according to the thickness of fascia or MTJ. The ROIs were positioned along the longitudinal section of the MGF, LGF and MTJ.

## Experimental design and protocol

### Measurement location

Only the dominant legs of participants were studied (25–27). Enrollees were asked to rest for 10 min before testing. In addition, they were asked to lie down in the prone position on the treatment bed, their feet were fully extended and slightly away from the bed, and their upper limbs were naturally placed on both sides of the body (26). When the knee was fully extended or flexed at 90°, the elasticity of the MGF, LGF and MTJ was quantified at 50° plantar flexion (PF50°), 0° (neutral position), and 20° dorsiflexion (DF20°) of the ankle joint. To fix the ankle and knee, customized and movable knee ankle foot orthoses were used. MGF and LGF were measured using tape measure at the muscle belly of the proximal 30% of the lower leg length, whereas MTJ was defined as the junction of MG tendon and AT (25–27), and a waterproof marker was used to determine the location of the measurement site. For better experimental measurement accuracy, participants were asked to refrain from high-intensity exercise for 48 h before testing, and they were asked to keep their bodies fully relaxed throughout the duration of testing.

### Procedures

**Figure 1:**
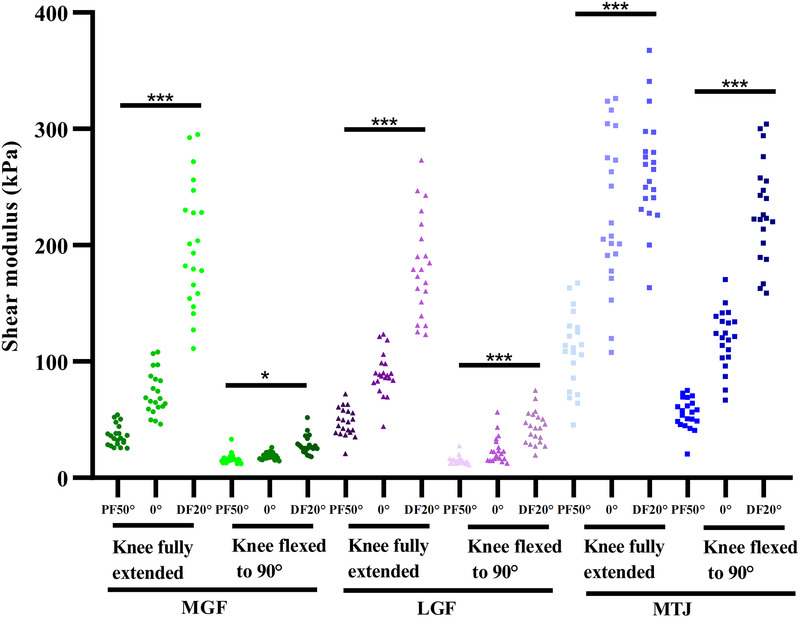
Typical maps of the elastic properties of MGF, LGF and MTJ in the longitudinal directions. MGF, medial gastrocnemius fascia; LGF, lateral gastrocnemius fascia; MTJ, myotendinous junction.

All participants received a USWE examination from physical therapists (L.C.Z and Z.J.P.) with 6 years of experience performing ultrasonography. In addition, the USWE examination was supervised by a sonographer (L.Y.Y) with 8 years of expertise. Shear elastic modulus was quantified using the AixPlorer ultrasonic scanner positioned on the skin markers at PF50°, 0° and DF20° with the knee plantarflexion (90° and 0° of knee joint angle, with 0° representing the fully extended knee). To ensure that the musculotendon restored its original elastic properties between angle switches, the shear elastic modulus at each ankle joint angle was measured at 5 min intervals (35). In addition, the measurement sequence for the knee joint and the ankle joint was as follows: the knee joint was first in the flexion position, then in the extension position, and the ankle joint was first at 50° plantar flexion, then at 0° neutral position, and finally at 20° dorsiflexion. According to our previous studies (25–27), first, sufficient ultrasound gel was applied on the skin markers. Second, the transducer midpoint was placed on the markers, and the B-mode was activated to ensure that the muscle belly was assessed, then rotated and oriented longitudinally until the gray-scale image displayed the appearance of the muscle or MTJ (Figure 1). Third, the mode of USWE was activated, the transducer was kept motionless for more than 8 s, and the image was frozen until the color in the ROI was uniform and several fibers were continuously visible (25–27). Three images were captured at each measurement site of muscle fascia or MTJ. Image quality was closely monitored throughout all measurements.

Two operators (L.C.Z. and Z.J.P.) took part in the inter-operator investigation, taking turns to measure each subject's MGF, LGF and MTJ over a 1-hour period and this was repeated by operator Z.J.P. with a 2 h interval. In the second test performed 5 days later, the same subjects participated at the same time, which was repeated by operator L.C.Z. for the intra-operator investigation. Subjects were asked to maintain their normal activity and avoid additional activity (26).

### Statistical analysis

Statistical analysis was performed using SPSS version 19.0 (SPSS, Chicago, IL). All data were presented as mean ± standard deviation. Data normality was tested by the Shapiro–Wilk test. Intra- and inter-operator reliability were evaluated by calculating the intraclass correlation coefficient (ICC). The intra-operator (measurements taken on 2 occasions separated by 5 days) and inter-operator (measurements by 2 operators) reliability were examined using ICC (3,1) and ICC (2,2). The standard error of the mean (SEM) was calculated by the formula SEM = standard deviation  ×   √(1−ICC), the coefficient of variance (CV) was calculated by the formula CV = (Standard deviation/mean)  ×  100%, while the minimal detectable change (MDC) was computed by the formula MDC = 1.96  ×  SEM  ×   √2. ICC values > 0.50 were considered as poor, 0.50–0.70 as moderate, 0.71–0.90 as good, and more than 0.90 as excellent. For the data on passive joint elastic properties, two-way analysis of variance (ANOVA) tests (ankle angle  ×   knee angle) with repeated measures were performed, followed by *post hoc* comparisons using two-sided, paired, Bonferroni-corrected *t* tests. comparisons between groups were made using independent samples *t*-tests. *P* < 0.05 was considered indicative of statistical significance.

## Results

### Intra- and inter-operator reliability of mean elastic modulus of MGF, LGF and MTJ

The ICC, MDC, CV, and SEM for the intra- and inter-operator reliability for the mean elastic modulus of the MGF, LGF and MTJ can be found in Tables 1, 2. The intra-operator reliability (ICC = 0.893−0.980) and inter-operator reliability (ICC = 0.907−0.981) were excellent for the elastic modulus of the MGF and LGF. The SEM (kPa) ranged from 0.79 to 20.07; the MDC (kPa) ranged from 2.19 to 55.62; and the CV (%) ranged from 15.76 to 60.45. The intra-operator reliability (ICC = 0.906−0.946) and inter-operator reliability (ICC = 0.921−0.976) were excellent for the elastic modulus of the MTJ. The SEM (kPa) ranged from 5.85 to 23.74; the MDC (kPa) ranged from 16.21 to 65.80; and the CV (%) ranged from 17.86 to 31.37.

**Table 1:** Intra-operator reliability of mean elastic modulus values of MGF, LGF and MTJ.

Measurement position	Knee angle	Ankle angle	Test 1 (kPa)	Test 2 (kPa)	MDC (kPa)	CV (%)	SEM (kPa)	ICC (95% CI)
MGF	Knee 0°	PF50°	36.21 ± 8.91	36.20 ± 7.51	6.58	24.61	2.37	0.980 (0.950–0.992)
		0°	72.88 ± 18.50	75.07 ± 19.39	17.01	25.83	6.14	0.943 (0.859–0.977)
		DF20°	199.63 ± 53.08	185.97 ± 40.71	55.05	26.59	19.86	0.942 (0.856–0.976)
LGF		PF50°	48.03 ± 12.02	48.70 ± 12.09	8.21	25.03	2.96	0.978 (0.945–0.991)
		0°	89.54 ± 18.30	87.17 ± 16.19	13.46	20.44	4.86	0.966 (0.916–0.986)
		DF20°	181.21 ± 42.57	167.29 ± 37.71	52.77	23.49	19.04	0.917 (0.797–0.966)
MTJ		PF50°	109.54 ± 32.73	112.70 ± 30.22	30.20	29.88	10.90	0.977 (0.943–0.991)
		0°	227.78 ± 65.84	215.65 ± 47.97	65.80	28.91	23.74	0.936 (0.843–0.974)
		DF20°	264.29 ± 46.29	247.64 ± 38.88	51.32	17.51	18.52	0.944 (0.863–0.977)
MGF	Knee 90°	PF50°	16.52 ± 4.49	17.82 ± 5.07	4.87	28.45	1.76	0.954 (0.888–0.981)
		0°	18.91 ± 2.98	20.54 ± 3.36	4.37	15.76	1.58	0.893 (0.736–0.957)
		DF20°	28.10 ± 7.98	27.91 ± 6.15	5.85	28.40	2.11	0.963 (0.910–0.985)
LGF		PF50°	14.81 ± 3.55	15.16 ± 3.46	2.20	23.97	0.79	0.974 (0.937–0.990)
		0°	23.67 ± 11.04	22.89 ± 9.82	6.12	46.64	2.21	0.970 (0.943–0.991)
		DF20°	43.31 ± 14.20	41.18 ± 12.40	11.81	32.79	4.26	0.959 (0.899–0.983)
MTJ		PF50°	55.46 ± 13.08	59.46 ± 11.41	16.21	23.58	5.85	0.910 (0.779–0.964)
		0°	119.57 ± 25.07	114.73 ± 25.53	26.48	22.25	9.55	0.946 (0.867–0.978)
		DF20°	229.20 ± 43.03	213.36 ± 43.75	51.99	20.51	18.76	0.906 (0.768–0.962)

MGF, medial head of the gastrocnemius muscle fascia; LGF, lateral head of the gastrocnemius muscle fascia; MTJ, musculotendinous junction.

PF50°, plantar flexion 50°; 0°, neutral position; DF20°, dorsiflexion 20°.

MDC, minimal detectable change; 95% CI, 95% Confidence Interval; CV, coefficient of variation; SEM, standard error in measurement; ICC, intra-class correlation coefficient.

**Table 2:** Inter-operator reliability of mean elastic modulus values of MGF, LGF and MTJ.

Measurement position	Knee angle	Ankle angle	Test 1 (kPa)	Test 2 (kPa)	MDC (kPa)	CV (%)	SEM (kPa)	ICC (95% CI)
MGF	Knee 0°	PF50°	36.21 ± 8.91	35.38 ± 7.07	6.20	19.98	2.24	0.947 (0.869–0.978)
		0°	72.88 ± 18.50	72.59 ± 19.24	17.01	26.51	6.14	0.981 (0.953–0.992)
		DF20°	199.63 ± 53.08	189.95 ± 47.39	55.05	26.59	19.86	0.978 (0.945–0.991)
LGF		PF50°	48.03 ± 12.02	48.67 ± 10.45	7.10	25.03	2.56	0.969 (0.923–0.987)
		0°	89.54 ± 18.30	84.46 ± 15.76	17.01	26.51	6.14	0.971 (0.930–0.988)
		DF20°	181.21 ± 42.57	179.16 ± 45.40	52.77	25.34	19.04	0.975 (0.939–0.990)
MTJ		PF50°	109.54 ± 32.73	104.49 ± 29.15	29.13	29.88	10.51	0.933 (0.835–0.973)
		0°	227.78 ± 65.84	216.00 ± 57.35	65.80	28.91	23.74	0.976 (0.941–0.990)
		DF20°	264.29 ± 46.29	268.82 ± 49.09	51.32	18.26	18.52	0.972 (0.930–0.989)
MGF	Knee 90°	PF50°	16.52 ± 4.49	16.82 ± 3.97	4.31	27.18	1.56	0.927 (0.820–0.970)
		0°	18.91 ± 2.98	19.11 ± 2.95	4.37	15.44	1.58	0.919 (0.801–0.967)
		DF20°	28.10 ± 7.98	27.87 ± 6.16	5.85	28.40	2.11	0.960 (0.902–0.984)
LGF		PF50°	14.81 ± 3.55	15.22 ± 2.62	2.20	23.97	0.79	0.907 (0.771–0.962)
		0°	23.67 ± 11.04	22.61 ± 7.57	6.12	46.64	2.21	0.938 (0.848–0.975)
		DF20°	43.31 ± 14.20	40.91 ± 11.73	11.81	32.79	4.26	0.964 (0.911–0.985)
MTJ		PF50°	55.46 ± 13.08	53.95 ± 12.49	16.21	23.58	5.85	0.965 (0.913–0.986)
		0°	119.57 ± 25.07	117.84 ± 23.52	25.06	20.97	9.04	0.921 (0.806–0.968)
		DF20°	229.20 ± 43.03	216.10 ± 38.92	51.99	18.77	18.76	0.940(0.851–0.975)

MGF, Medial head of the gastrocnemius muscle fascia; LGF, Lateral head of the gastrocnemius muscle fascia; MTJ, musculotendinous junction.

PF50°: plantar flexion 50°; 0°: neutral position; DF20°: dorsiflexion 20°.

MDC, minimal detectable change; 95% CI, 95% confidence interval; CV, coefficient of variation; SEM, standard error in measurement; ICC, intra-class correlation coefficient.

### Effect of different knee joint angles on elasticity of MTJ, MGF and LGF

The relationships between passive knee angle and muscle fascia elastic properties are shown in Table 3. The elasticities of the MTJ, MGF and LGF with the knee flexed to 90° were significantly lower than that with the knee fully extended (At DF20°, the *p*-value associated with MTJ was 0.012, whereas all other *p*-values were below 0.001).

**Table 3:** Comparison of the elastic properties between the knee fully extended and the knee flexed to 90°.

Measurement position	Ankle angle	Knee 0°	Knee 90°	Cohen's *d*	*P* value
MGF	PF50°	31.16 ± 8.95	11.5 ± 4.49	2.85	0.000
	0°	67.83 ± 18.57	13.9 ± 2.98	4.06	0.000
	DF20°	194.64 ± 53.08	23.1 ± 7.98	4.52	0.000
LGF	PF50°	42.98act±12.07	9.79 ± 3.54	3.73	0.000
	0°	84.5 ± 18.32	18.76 ± 11.34	4.32	0.000
	DF20°	177.14 ± 44.87	38.31 ± 14.2	4.17	0.000
MTJ	PF50°	104.5 ± 32.78	50.45 ± 13.08	2.17	0.000
	0°	222.77 ± 65.84	114.57 ± 25.08	2.17	0.000
	DF20°	259.27 ± 46.3	219.43 ± 54.33	0.79	0.012

MGF, medial head of the gastrocnemius muscle fascia; LGF, lateral head of the gastrocnemius muscle fascia; MTJ, musculotendinous junction.

PF50°, plantar flexion 50°; 0°, neutral position; DF20°, dorsiflexion 20°.

### Association of elastic properties of MGF, LGF and MTJ with passive ankle joint angle

**Figure 2:**
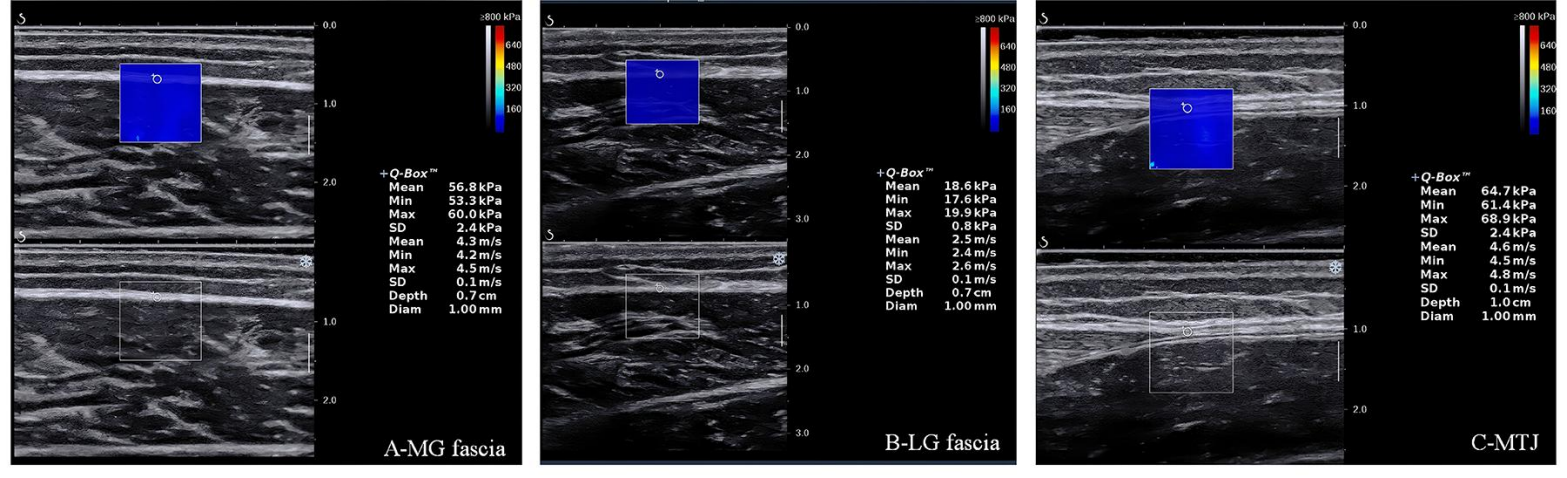
Variations in the elasticity of MGF, LGF and MTJ at PF50°, 0°, and DF20° of passive ankle joint with the knee fully extended/flexed at 90°. *P < 0.05; ***P < 0.001; NS: non-significant at *P* > 0.05.

The relationships between passive ankle angles and muscle fascia elastic properties are shown in Figure 2. Regardless of knee position, the elasticity of the MTJ, MGF and LGF increased as ankle dorsiflexion increased (MGF: F = 139.301, *p* < 0.001, partial *η*^2^ = 0.877; LGF: F = 66.651, *p* < 0.001, partial *η*^2^ = 0.774; MTJ:F = 9.054, *p* = 0.001, partial *η*^2^ = 0.317; All *p*-values < 0.001).

**Figure 3:**
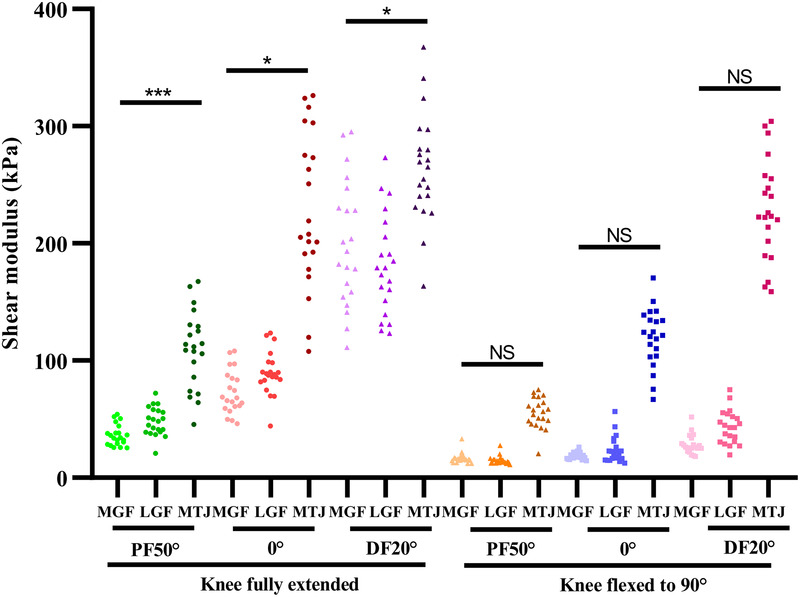
Variations in the elasticity of MGF, LGF and MTJ at the same angle of passive ankle joint with the knee fully extended/flexed at 90°. *P < 0.05; ***P < 0.001; NS, non-significant at *P* > 0.05.

At the same knee and ankle joint angle, the differences among three muscle fasciae were shown in Figure 3. Regardless of the ankle and knee angles, the elastic properties of the MTJ were always higher than those of the MGF and LGF (All *p*-values < 0.001). When the knee joint was fully extended, regardless of ankle position, there was a significant difference between MGF elasticity and LGF elasticity. The elasticity of the LGF at 0° and PF50° was significantly greater than that of the MGF (PF50°: *p* < 0.001, 0°: *p* = 0.006), while elasticity was higher in the MGF than in the LGF at DF20° (*p* = 0.011). When the knee joint was flexed at 90°, there was no significant difference in the elasticity of the MGF compared to the LGF, regardless of the ankle joint angle (PF50°: *p* = 0.222, 0°: *p* = 0.178, DF20°: *p* = 0.063).

### Discussion

Regarding the main findings of this study, first, the USWE exhibited excellent reliability in measuring the elastic modulus of the MGF, LGF, and MTJ. Second, the elasticity of the MTJ, MGF and LGF with the knee flexed to 90° was significantly lower than that with the knee fully extended. Third, regardless of knee position, the elasticity of the MTJ, MGF and LGF increased with increasing ankle dorsiflexion. Fourth, the elastic property of the MTJ was always higher than that of the MGF and LGF regardless of ankle and knee angles. Finally, when the knee joint was fully extended, the elasticity of the LGF at 0° and PF50° was significantly greater than that of the MGF, while it was higher in the MGF than in the LGF at DF20°. These findings demonstrate that force transmission occurs not only at the myotendinous junction but also via pathways of muscle fascia and the MTJ. Furthermore, *in vivo* muscle force transmission is not independent. Although several pathways exist for muscle force transmission, such as tendons and muscle fascia, the relative elasticity of the soft tissues involved in each pathway may determine the fraction of force transmitted.

### Reliability of elasticity measurement of MTJ, MGF and LGF at different knee and ankle joint angles

This study demonstrated that USWE exhibits excellent intra- and inter-operator reliability for measuring the elastic modulus of the MGF, LGF, and MTJ. The findings are consistent with previous research on SWE reliability in musculoskeletal tissues. Studies on other muscle groups have reported comparable high intra-operator ICCs. For instance, Maïsetti et al. (36) reported excellent intra-operator reliability (ICC = 0.97) for SWE measurements of the gastrocnemius muscle belly at rest, which aligns with the high reliability we observed in the fascial tissues of the same muscle. Furthermore, research on tendons has shown similar trends. Aubry et al. (37) found excellent intra- and inter-observer reproducibility for the SWE assessment of the Achilles tendon (ICCs > 0.90), corroborating our high-reliability results for the MTJ, a transitional tissue between muscle and tendon. The slightly lower (yet still good-to-excellent) ICCs in some positions, particularly during plantarflexion or at the knee 90° position, may be attributed to the altered tension and depth of the tissues, which can influence shear wave propagation. Notably, even under these potentially challenging conditions, the ICCs remained largely above 0.90, underscoring the robustness of the USWE technique. This context-dependent variability has also been noted by Eby et al. (38), who emphasized that muscle stiffness measurements are highly posture-dependent yet reliable when standardized.

### Effect of knee joint angle on elasticity of MTJ, MGF and LGF

The present study demonstrated the dependence of the elastic properties of muscle-fascia and MTJ on the level of knee and ankle extension angle. Based on our literature search, this is the first study to delineate the mechanical behavior of the MTJ and muscle-fascia in relation to passive ankle dorsiflexion. The elasticity of the MTJ, MGF and LGF with the knee flexed at 90° was significantly lower than that with the knee fully extended, and the elasticity of these structures enhanced with increasing ankle dorsiflexion. No studies have previously estimated the changes in elasticity of the muscle-fascia and MTJ at different knee and ankle joint angles. Yet, some previous studies have reported changes in the elasticity of MG, LG and SOL during ankle and knee dorsiflexion. For example, Hug et al. (39) used supersonic shear imaging to assess the shear elastic modulus of the MG during passive knee plantarflexion (0° and 90°), and their results showed that the shear elastic modulus of the MG at 0° was higher than that at 90°. The findings of Le et al. (40) also revealed that the shear elastic modulus of the MG at 0° was higher than that at 90°. In addition, both studies found that the shear elastic modulus of MG and LG increased with passive ankle dorsiflexion (39, 40). Thus, when the lower limb is subjected to sufficient mechanical stress, the passive motion of the joint will significantly change the elasticity of the muscle-fascia and MTJ. Therefore, we speculated that passive flexion of the knee and ankle joints can not only change the elasticity of muscles and tendons but also increase the elasticity of fascia and MTJ. The findings can be explained by both anatomical factors and the stretching effect. From the anatomical aspect, the MG and LG arise from the femoral condyles (cross the knee joint), and their muscle-fascia extends downward to form MTJ with SOL-fascia, i.e., the starting point of the Achilles tendon, and ends at the calcaneal tuberosity (41). Thus, the muscle fascia and MTJ are affected by the angle of the knee and ankle joints. In addition, the increased elastic properties observed in the muscle's fascia and MTJ were as expected (41). The anatomical characteristics showed that the soft tissue of the lower limb becomes significantly stretched with increasing passive ankle dorsiflexion. Muscle fasciae, which are in series with inter-muscles and intra-muscles, also play a role, with previous studies concluding that they contribute to elastic energy storage during passive stretching (42). Indeed, the elastic energy storage and recovery capacity of muscle fascia and MTJ can be improved by stretching, such as passive ankle dorsiflexion or passive knee flexion (42, 43). The present findings revealed that the elasticity of the MGF, LGF and MTJ increases with ankle dorsiflexion or knee extension, suggesting that the muscle fascia and MTJ can act as a spring, not only contributing to muscle and myofascial force transmission and elastic energy storage but also playing an important role in maintaining lower limb stability (44). These findings seem plausible, as such deformations of the muscle fascia and MTJ match and optimize muscle flexibility, thereby comprising a functional entity of muscle fascia.

### Association of elastic properties of MGF, LGF and MTJ with passive ankle joint angle

This study also showed that regardless of the ankle and knee angles, the elasticity of MTJ was always higher than that of MGF and LGF, similar to previous studies (45). Yoshida et al. examined the elastic properties of MTJ aponeurosis (center of the MTJ, proximal of the MTJ and distal of the MTJ) at 15° knee flexion and 15° ankle plantar flexion. The results indicated that the elasticity of MTJ-Proximal and MTJ-Central were significantly smaller than that of MTJ-Distal, while MTJ-Proximal was significantly smaller than that of MTJ-Central (45). Changes in myofascial elasticity during the passive stretching of the lower limb (including knee joint and ankle joint) reflect the physiologic characteristics of muscle fascia and MTJ interaction. The ability of muscle to generate and transmit mechanical force is related to interactions between the muscle fiber type and elastic structures associated with its myofascial structures and tendons (46). According to the balance between the metabolic cost of muscle contraction and energy produced by elastic deformation, the key mechanism to improve walking energy expenditure is to maintain muscle and myofascial tension in an optimal state (47). Regardless of the ankle and knee angles, in this study, the elasticity of MTJ was always higher than that of muscle fascia. The elasticity differences of muscle fascia and MTJ under different knee and ankle flexion angles are related to their own physiological structure. From an anatomical point of view, the function of MTJ is to transmit the skeletal muscle strength to the adjacent tendon, thus it is subjected to higher mechanical stress than muscle fascia, resulting in greater elastic deformation (48). This explains why the muscle strain of the gastrocnemius tends to occur at the MTJ (48). It has been reported that the recurrence rate of muscle strain is approximately 13%–23% (41, 47, 49). A higher stiffness (elastic properties) of the junction between the muscle and aponeurosis (i.e., MTJ) is considered to be one of the main risk factors of muscle strain (49). However, stiffness (elastic properties) is commonly evaluated subjectively by palpation, with no independent and objective method to measure the elasticity of muscle, muscle fascia and MTJ separately. Combined with our previous results (25–27), the USWE can accurately examine localized changes in the elastic properties of gastrocnemius muscle and Achilles tendon in healthy subjects and individuals with plantar fasciitis. On the basis of these results, we believe that USWE can be utilized to evaluate muscle, muscle fascia and MTJ before and after daily exercise training, even after muscle injury (50). Furthermore, a meta-analysis has reported that a predictable link exists between tendon strength and elastic properties, and clinicians could reasonably predict the ultimate stress or failure properties of tendons through the elastic properties measured via ultrasound examination. Therefore, we speculate that the stiffness evaluated by USWE not only can potentially be used as an objective predictor of the efficacy and time of injury recovery but also to determine the appropriate period to return to exercise, thus preventing the recurrence of muscle injury. However, the above conjecture still needs to be confirmed by further studies.

Since MTJ is a common injury site (48), the relative elastic properties of the muscle-fascia and MTJ may determine the fraction of force transmission through them. The integrity of MTJ is not only essential to permit muscle strength transmission but also to optimize energy expenditure during different functional tasks, such as running and jumping. If the elastic properties of MTJ change, the muscle, its fascia and tendon will be subjected to greater mechanical stress, thereby increasing the risk of muscle, myofascial or tendon injury. Therefore, we suggest that a warm-up is performed before the process of functional activities, as it not only activates the muscle and fascia first to reduce the risk of injury during exercise, but also improves the explosive power and tolerance of muscle and fascia. After training or exercise, USWE can be used to evaluate the elasticity of MTJ to determine whether it needs to relax and return to normal level in a short period.

This study presents a novel finding regarding the elasticity differences between MGF and LGF. When the knee joint is fully extended, the elasticity of the LGF at 0° and PF50° is significantly greater than that of the MGF, while the elasticity is higher in the MGF than in the LGF at DF20°. However, when the knee joint is flexed at 90°, there is no significant difference between the elasticity of MGF and LGF. This means that the activation level of MGF and LGF is not identical during passive ankle dorsiflexion or passive knee flexion, confirming our initial hypothesis. When the knee is flexed at 90°, fascial traction on both the MGF and LGF is relatively reduced, resulting in lower mechanical stress and a decreased likelihood of strain injury under passive or low-load conditions. This aligns with the observed absence of significant elasticity differences between MGF and LGF at this joint angle, suggesting a more balanced load-sharing mechanism during knee flexion. In contrast, during explosive movements involving simultaneous knee extension from a flexed position and rapid ankle dorsiflexion from plantarflexion, the MGF assumes a greater proportion of force transmission compared to the LGF. This can be attributed to the anatomical and functional predominance of the MG in forceful plantarflexion, as evidenced by its larger cross-sectional area, greater volume and higher force contribution-exceeding 70% during plantarflexion tasks (51). Besides, the smaller rotation angle of the MG relative to the LG (52) may allow the MGF to experience higher tensile loads during fast, multi-joint movements, where the muscle-tendon unit undergoes rapid lengthening and high strain rates. Therefore, in both warm-up routines and post-activity screening, particular attention should be paid to the integrity and functional status of the MGF: the proactive assessment of its elasticity, stiffness and susceptibility to overload may facilitate early detection of susceptibility to strain, especially in athletes or individuals engaged in activities requiring explosive lower-limb movements.

This study underscores the excellent reliability of USWE in the *in vivo* assessment of the gastrocnemius fasciae and MTJ and is the first to systematically map the dynamic regulation of their elasticity by passive knee and ankle joint angles. The findings provide crucial imaging-based biomechanical evidence with direct implications for understanding sports-related muscle strain injuries. The significantly higher elasticity of the LGF during knee extension and ankle plantarflexion (e.g., mimicking the push-off phase in sprinting and jumping) suggests that this region may sustain greater mechanical stress in such postures, offering a potential biomechanical explanation for the clinical observation of the lateral head of the gastrocnemius being particularly susceptible to strain during high-velocity activities (53). Conversely, the higher elasticity of the MGF at ankle dorsiflexion may indicate its distinct mechanical role in postures requiring deep ankle flexion (e.g., deep squats). Moreover, the consistently higher elasticity of the MTJ across all postures underscores its critical role in force transmission, where alterations in its elastic state could be linked to overuse-related injuries (8). Consequently, quantifying fascia and MTJ elasticity in functional postures via USWE could emerge as a non-invasive tool to profile the “stiff” or “compliant” state of an individual's myofascial unit. This profiling holds promise for identifying athletes at an elevated injury risk, developing personalized stretching or strengthening regimens, and providing objective imaging metrics for monitoring rehabilitation progress.

This study has several potential limitations. First, only healthy physically active adults were recruited, hence further studies need to be conducted to evaluate the myofascial elasticity distribution in patients with muscle injury. Second, elasticity values between males and females were not compared because the minimum Q-box diameter of USWE is 1 mm, while the myofascial thickness of most females was less than 1 mm in preliminary experiments. Therefore, the findings of this study cannot be applied to female subjects. Third, we did not use EMG to monitor MG and LG activity to ensure whether the muscles contracted during the entire experiment. Instead, every participant was asked to remain relaxed, and there was no sign of muscle contraction on real-time ultrasound images. Thus, we believe that each subject followed the oral instructions and the relaxed muscle criterion was fulfilled. Fourth, there are constraints in positional control. The extensive collection of measurement data and images necessitated prolonged time for each participant during the assessment phase, which resulted in fatigue and slight involuntary posture shifts in participants' posture.

## Conclusion

This study demonstrates that USWE is a reliable tool for quantifying the elastic modulus of the gastrocnemius fascia (medial and lateral) and the MTJ, showing excellent intra- and inter-operator agreement (ICC > 0.89). Our experiment showed that the elasticity of the myofascial system is dynamically regulated by joint position, decreasing with knee flexion and increasing with ankle dorsiflexion, while the MTJ exhibited consistently higher elasticity than the fascial layers across all tested postures. Furthermore, significant differences in elasticity between the medial and lateral gastrocnemius fasciae were only observed with the knee fully extended and they varied with ankle angle, whereas no such difference was found at 90° of knee flexion. These findings provide important imaging-based biomechanical insights for understanding the mechanisms of exercise-related muscle injury and inform targeted rehabilitation strategies.

## Data Availability

The data that support the findings of this study are available on request from the corresponding author.
